# Autobiographical memory in semantic dementia: A longitudinal fMRI study

**DOI:** 10.1016/j.neuropsychologia.2009.08.020

**Published:** 2010-01

**Authors:** Eleanor A. Maguire, Dharshan Kumaran, Demis Hassabis, Michael D. Kopelman

**Affiliations:** a Wellcome Trust Centre for Neuroimaging, Institute of Neurology, University College London, 12 Queen Square, London WC1N 3BG, UK; b King's College London, Institute of Psychiatry, based at the Academic Unit of Neuropsychiatry, 3rd Floor, Adamson Centre, South Wing, St Thomas's Hospital, London SE1 7EH, UK

**Keywords:** Autobiographical memory, Semantic dementia, fMRI, Longitudinal

## Abstract

Whilst patients with semantic dementia (SD) are known to suffer from semantic memory and language impairments, there is less agreement about whether memory for personal everyday experiences, autobiographical memory, is compromised. In healthy individuals, functional MRI (fMRI) has helped to delineate a consistent and distributed brain network associated with autobiographical recollection. Here we examined how the progression of SD affected the brain's autobiographical memory network over time. We did this by testing autobiographical memory recall in a SD patient, AM, with fMRI on three occasions, each one year apart, during the course of his disease. At the outset, his autobiographical memory was intact. This was followed by a gradual loss in recollective quality that collapsed only late in the course of the disease. There was no evidence of a temporal gradient. Initially, AM's recollection was supported by the classic autobiographical memory network, including atrophied tissue in hippocampus and temporal neocortex. This was subsequently augmented by up-regulation of other parts of the memory system, namely ventromedial and ventrolateral prefrontal cortex, right lateral temporal cortex, and precuneus. A final step-change in the areas engaged and the quality of recollection then preceded the collapse of autobiographical memory. Our findings inform theoretical debates about the role of the hippocampus and neocortical areas in supporting remote autobiographical memories. Furthermore, our results suggest it may be possible to define specific stages in SD-related memory decline, and that fMRI could complement MRI and neuropsychological measures in providing more precise prognostic and rehabilitative information for clinicians and carers.

## 1 Introduction

Semantic dementia (SD) is a variant of fronto-temporal dementia. This progressive pathology is characterised initially by asymmetric atrophy of the anterior temporal lobes (Hodges & Patterson, 2007). It involves a range of symptoms that includes anomia, deterioration of expressive and receptive vocabulary, and a deficit in semantic memory (Hodges & Patterson, 2007; Hodges, Patterson, Oxbury, & Funnell, 1992; Snowden, Goulding, & Neary, 1989). Whilst these features are typically observed, there is less agreement about whether memory for personal everyday experiences, autobiographical memory, is compromised.

SD patients have been reported to show better preservation of recent relative to remote autobiographical memories (Graham & Hodges, 1997; Graham, Kropelnicki, Goldman, & Hodges, 2003; Hou, Miller, & Kramer, 2005; Nestor, Graham, Bozeat, Simons, & Hodges, 2002; Piolino et al., 2003; Snowden, Griffiths, & Neary, 1996). This pattern has been interpreted as support for the standard consolidation model of memory (Squire, 1992), where remote memories, dependent on the neocortex, are impaired because of the deleterious effect of SD on the integrity of temporal neocortex. By contrast their preserved recent memories have been held to reflect the relatively normal operation of the hippocampus in the early stages of the disease, or more uniform impairments across all time periods later in the disease. As such, SD patients are suggested to complement amnesic patients with selective hippocampal damage who are reported to have impaired recent but intact remote autobiographical memories (Hodges & Patterson, 2007; Nestor et al., 2002). The reverse temporal gradient observed in SD patients has been questioned, however, as findings from other studies have failed to confirm such a pattern, and instead report preserved recent and remote autobiographical memories in the early stages of the disease (McKinnon, Black, Miller, Moscovitch, & Levine, 2006; Moss, Kopelman, Cappalletti, De Mornay Davies, & Jaldow, 2003; Westmacott, Leach, Freedman, & Moscovitch, 2001). The lack of consistent findings may reflect the limited number of autobiographical memories tested in some studies (as few as 5 – McKinnon et al., 2006), the difficulty of assessing retrieval in SD where expressive speech can be variably compromised, and the stage in the disease process at which patients are tested (Matuszewski et al., 2009). The integrity of the hippocampus also needs to be considered, as not only lateral and anterior neocortical tissue is compromised in SD, but the hippocampus and medial temporal lobes are also affected to a variable degree even early in the course of the disease (Chan et al., 2001; Galton et al., 2001). Thus, as with cases of amnesia, the status of autobiographical memory in SD and the neural substrates that support it remain controversial.

In healthy participants our understanding of the neural basis of autobiographical memory has been enhanced by the use of fMRI, which has helped to delineate the brain networks involved and the response profile of the hippocampus. Recollecting memories of personal past experiences has been shown to rely on a distributed set of brain regions that includes the hippocampus (more often on the left), parahippocampal gyrus, lateral temporal cortices, posterior parietal cortex, retrosplenial cortex, posterior cingulate cortex, precuneus, thalamus, the medial prefrontal cortex, and cerebellum (Maguire, 2001; Svoboda, McKinnon, & Levine, 2006). These findings are highly consistent across studies (Svoboda et al., 2006), are evoked by verbal (e.g. Maguire & Frith, 2003) or photographic (Gilboa, Winocur, Grady, Hevenor, & Moscovitch, 2004) stimuli, and are robust even for single participants (Maguire, Vargha-Khadem, & Mishkin, 2001). Moreover, the majority of fMRI studies of autobiographical memory retrieval have documented hippocampal involvement irrespective of whether memories were formed recently or remotely in the distant past (Svoboda et al., 2006). By contrast, whilst there are numerous reports of structural MRI and resting state FDG-PET and SPECT in SD (e.g. Desgranges et al., 2007; Diehl et al., 2004; Nestor, Fryer, & Hodges, 2006), there is a dearth of fMRI memory studies involving SD patients.

In patients with brain lesions, several investigations have emphasised the contribution of right-sided lateral temporal and frontal lobe pathology in producing retrograde amnesia (Bright et al., 2006; Buccione, Fadda, Serra, Caltagirone, & Carlesimo, 2008; Kopelman, Stanhope, & Kingsley, 1999; Markowitsch et al., 1993; O’Connor, Butters, Miliotis, Eslinger, & Cermak, 1992; Ogden, 1993). This partial discrepancy with the fMRI findings in healthy participants, where left-sided activations are more prominent (Maguire, 2001; Svoboda et al., 2006), makes it interesting to know what would happen to fMRI activity patterns during autobiographical recollection when the hippocampi and temporal neocortex, particularly on the left, are atrophied. One possibility is that residual hippocampal tissue would still be activated during autobiographical retrieval, or that right fronto-temporal or other cortical regions would be recruited if the hippocampi were malfunctioning.

Here we examined whether or not there was a temporal gradient in autobiographical memory recall in the context of SD, as have previous studies. However, we extended this previous work in a number of ways. Given the well-established brain network known to support autobiographical memory retrieval in fMRI studies of healthy participants (Maguire, 2001; Svoboda et al., 2006), we sought to ascertain the effect of SD on this network. Would the remnant tissue in regions of atrophy be active? Would there be evidence of compensatory mechanisms? Moreover, as well as contributing novel insights from a ‘snapshot’ view of SD at one point in time, we examined how the progression of SD affected the brain's autobiographical memory network. We did this by testing an SD patient with fMRI on three separate occasions, each one year apart, during the course of his disease. In this way we hoped to provide new information to aid the understanding of the effect of SD-related temporal lobe atrophy on autobiographical memory, and to explore the mechanisms of change through time, a relatively neglected topic to date but one of prognostic and theoretical significance. Overall, therefore, our findings might provide important information for clinicians and carers, whilst also informing key theoretical debates about the role of the hippocampus and neocortical areas in supporting remote autobiographical memories.

We charted the effect of SD on the autobiographical memories of patient AM. He was in effect his own control as we compared his performance over successive years. We employed a paradigm similar to that used by Gilboa et al. (2004) who tested healthy participants. As well as activating the classic autobiographical memory network, this paradigm had a number of advantages for our purpose. First, the stimuli were selected without the knowledge of the participant, in our case by AM's wife of fifty years. Second, the stimuli were photographs, which reduced reliance on AM's compromised verbal skills. Longitudinal testing in this case was only possible because of the unusually large reservoir of photographs accrued by AM's family over many decades. Thus during each year's fMRI scan we were able to test recall for many different autobiographical memories (i.e. 75 unique events in total over the course of the study, none of which was repeated), ranging from recent events to those that had occurred remotely in the past.

## 2 Methods

### 2.1 Case history

AM, a 70-year-old right-handed retired merchant seaman with 9 years of formal education, was first seen in the St Thomas's Neuropsychiatry and Memory Disorders Clinic in November 2001. At that time AM and his wife reported progressive memory loss over the last 2–3 years, particularly involving remembering peoples names, difficulty in word-finding, and difficulties in comprehension such that AM would occasionally “go blank” in conversation. He also had some difficulty with conceptual tasks, such as following instructions, but he could do home repairs using well-practised skills. AM reported that he had great difficulty in remembering telephone messages, but memory for day-to-day episodes was relatively well preserved. AM had noticed that he sometimes recalled the names of people and objects after a long latency period. There was no known family history of dementia. AM had been treated for depression on two occasions in the past but was not depressed during this study.

During this initial presentation, AM was fully orientated in time and place and could give an excellent account of recent news events, e.g. the then recent invasion of Afghanistan, although he could not remember the name of the World Trade Centre. He knew that he had been to France on a day trip the week before, and he was able to describe accurately what he had done there. He was able to name high frequency items, such as jacket, sleeve, cuff, and watch, but not low frequency items such as a lapel, watch face, buckle, or skirting board. On formal neuropsychological testing AM showed relatively well preserved IQ and executive function, as measured on the Modified Card-sorting test (Table 1). However, he could name only 4 items correctly out of 30 on the Graded Naming Test. He scored poorly on a verbal memory test for stories (WMS-R Logical Memory) possibly reflecting his semantic and language difficulties, whilst on the WMS-R Visual Reproduction subtest he scored at the 72nd percentile for immediate recall, and at the 66th percentile for delayed recalled. On the Recognition Memory Test he recognised 41 out of 50 words correctly, and 37 out of 50 faces. On FAS verbal fluency AM scored 27. Further investigations revealed preserved reading of regular and irregular words and non-words, and preserved mental calculation (see Cappelletti, Butterworth, & Kopelman, 2006). On the Autobiographical Memory Interview (Kopelman, Wilson, & Baddeley, 1990), he showed a recency effect in recalling personal semantic facts and a U-shaped curve in retrieving autobiographical incidents.

Standard blood tests were normal, and an MRI brain scan showed focal atrophy of the left temporal lobe involving the lateral, inferior and medial temporal structures including the left hippocampus. There was only minor atrophy in the right temporal lobe and at the frontal poles. A diagnosis of progressive fluent aphasia was made within the context of a fronto-temporal (‘semantic’) dementia, involving predominantly focal left temporal lobe atrophy.

AM was monitored over the next 6 years – Table 1 summarises his neuropsychological test scores. His mental state fluctuated somewhat, and he would tend to be more confused in the early morning and in the late evening than in the middle of the day. Over the course of 2002, there was a slow deterioration, and Mrs AM noticed that her husband had increasing difficulty in understanding certain words such as ‘garden’ and ‘hobby’. She also reported that his conversation had become more stereotyped, and he liked to keep to familiar themes and repetitive stories (usually about football matches). Otherwise he would tend to go silent in company. However, AM retained excellent recall of recent events. In 2003, his wife reported further deterioration, and AM would commonly comment “I can’t remember what that means.” He often had difficulty understanding conversation, when something was said for the first time, and he often repeated himself. He made a number of interesting semantic errors saying “radio rings” for telephone, “rubbish plants” for weeds, “letters” for words, “bread” for potatoes, “company” for football team, and “branches” for flowers. Mental calculation remained well preserved. A trial of an anticholinesterase agent at this time had minimal benefits. In 2004, the naming difficulty became more pronounced and impairments in comprehension more evident, although AM's episodic memory appeared well preserved, e.g. in describing the rugby World Cup final. AM was having increasing difficulty remembering names, and also now in recognising the faces of his friends. Further words that he could no longer comprehend included ‘scalp’, ‘nutrition’, ‘orbit’, ‘pod’, ‘snarl’ and ‘predatory’. However, AM was still able to carry out procedural skills, and he redecorated and rewired his kitchen at this time. During 2005, AM's deterioration accelerated. Although his jocular manner and sociable personality was still much in evidence, AM became more withdrawn with increased word-finding difficulties, paraphasias, and comprehension problems. Moreover, his procedural skills had deteriorated, and AM had not managed to repair a leak in the loft that he would have coped with in the past, and he had lost interest in repairing his car. On the other hand, this appeared to be mainly a problem in planning and organisation — when a plumber came to repair the leak, AM saw what needed to be done and he then got into action, assisting the plumber in a completely capable fashion. Repeat neuropsychological testing during this time showed deterioration on the Graded Naming Test, together with parallel impairments on the British Picture Vocabulary Scale, and the PALPA Word-Picture Matching task. By October 2005, four months after the final fMRI scan of this study, AM scored 0 on the Graded Naming Test.

#### 2.1.1 Control participants

Ten right-handed healthy males also participated in the study: mean age 66.7 years (S.D. 3.2), mean number of years retired 7.17 (S.D. 5.65), mean VIQ 109.7 (S.D. 6.93); no differences between AM and the controls on any of these measures (all *p* > 0.2). For the analysis of structural MRI brain scans, AM was compared with the group of 10 control participants. Only one of the control participants had suitable photographic stimuli for the fMRI experiment. He was 69 years old, had been retired 6.5 years, and had a VIQ of 112.

### 2.2 The present study

AM attended on three separate occasions for fMRI and structural MRI investigations: in March 2003, April 2004, and June 2005. In this longitudinal investigation AM was in effect his own control. However, we also tested one of the healthy control participants (see details above) to establish that the expected ‘classic’ pattern of activations associated with autobiographical memory retrieval was evoked by the current task. AM (and his wife) and the control participants gave informed written consent to participation in the study in accordance with the local research ethics committee.

**Fig. 1:**
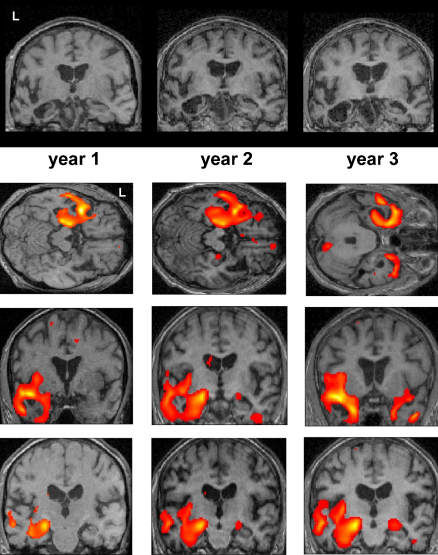
Structural MRI brain scans of the patient. Top panels show coronal sections through the brain of patient AM at the level of the mid-temporal lobe for each year. Running vertically below each year's coronal section are further axial and coronal sections from that year's MRI scan superimposed on which are the results of the VBM analysis for that year. The pronounced atrophy of cortical and medial left temporal regions is apparent at year 1. Scans from the subsequent years show a progression in this atrophy, and additional pathology starting in homologous areas on the right. See main text, and Table S1 (in Supplementary Materials) for full details of the VBM findings.

Fig. 1 shows the progressive change in AM's structural MRI brain scans during the study. In order to formally assess the extent of atrophy in patient AM across the whole brain, we compared the structural MRI scans of AM with those of the group of 10 control participants using voxel-based morphometry (VBM; Ashburner & Friston, 2005; see also Section 2.5). This revealed that at year 1, AM had significantly less grey matter volume in the left hippocampus and left anterior-lateral temporal lobe. There were no grey matter differences anywhere else in the brain. By year 2, the atrophy had spread and was starting to involve the right anterior temporal cortex, the right temporal pole, right anterior hippocampus, and right cerebellum. By year 3, there was very significant atrophy of both temporal lobes, including the hippocampi, still more extensive on the left, and also the right cerebellum, with no other significant grey matter differences evident elsewhere in the brain. See Table S1 (Supplementary Materials) for full details of the VBM findings.

### 2.3 Stimuli

Fortunately there was a long history in AM's family of taking photographs of memorable occasions. This exceptionally large photographic collection formed the basis of the stimuli for the longitudinal assessment of AM's autobiographical memory. Photographs were selected in consultation with AM's wife of 50 years, without the involvement of AM himself. Thus, the only time AM saw the photographs was when he performed the tasks in the scanner. His wife selected photographs that depicted specific events or occasions, that she thought her husband should have a high likelihood of recalling, and that she was sure he had not looked at either at all since they were taken, or for a considerable time. Whilst we cannot rule out the possibility that AM had, unbeknownst to his wife, examined the photographs more recently, Mrs AM thought this was highly unlikely and indicated that since the onset of his illness several years previously, AM had lost interest in taking and looking at family photographs. Photographs included those from AM's childhood and youth, to which his wife also had access and was able to provide full information about the events depicted. His wife ensured that AM did not access any of the photographic stimuli used in the study prior to the experiments. The stimuli for each year's experiment were different.

During each fMRI session, 25 photographs depicting AM's autobiographical memories were shown, thus 75 autobiographical photographs in total over the three years. Each set of 25 photographs comprised stimuli that ranged from the 1930s to one year prior to a scan, thus spanning eight decades with approximately three stimuli per decade. AM appeared in about 50% of the photographs, which were evenly distributed across the decades within a scan set, and also across the three scan sets. Stimuli were in a mixture of landscape and portrait formats shown in black and white, on a black background, filling 90% the screen. For every one of AM's autobiographical photographs, a ‘foil’ photograph was included that was both visually and semantically very similar, but unknown to AM. Each foil was presented in the same format as its companion autobiographical photograph. This foil condition controlled for high level visual processing, imagery, and general semantic retrieval, but only the autobiographical photographs referred to specific events in AM's life. A third stimulus type was included as a low level control condition. Each of the autobiographical stimuli was run through a computer programme which scrambled it, and rendered the overall picture meaningless. The use of these low level control stimuli made it possible to examine the wider brain network associated with autobiographical memory, the primary interest of the study. Stimuli for the control participant were prepared in exactly the same manner in consultation with his wife — his stimuli ranged from the 1930s to one year prior to a scan, thus spanning eight decades with approximately three stimuli per decade. He too appeared in about 50% of the photographs, which were evenly distributed across the decades; he had not looked at the photographs for many years; and for every one of the control's autobiographical photographs, a ‘foil’ photograph was included that was both visually and semantically very similar, but unknown to the participant.

For AM's first visit in year 1, a fourth condition was also included. The stimuli were photographs of famous public events that spanned a similar timescale to his autobiographical events. The intention with this condition was to assess retrieval of complex events but ones that were not as self-relevant and that were more semanticised. However, as AM performed relatively poorly (see Section 3), this condition was not included in the subsequent years’ experiments. This is because when a patient performs poorly on a task, or is guessing, it makes any ensuing brain activations difficult to interpret (Price, Crinion, & Friston, 2006).

### 2.4 Task and procedure

On each of the three occasions of scanning, the procedure was the same for AM and also for the control participant. Extensive training was provided on all tasks and procedures before scanning, with additional practice in the scanner prior to the main experimental sessions. During scanning there were three runs (four for patient AM in year 1 as he also attempted the public events task), each lasting just over 10 min, with several minutes break between each run whilst the participant remained in the scanner. For each visit, in total there were 25 autobiographical trials, 25 matched foil trials, and 25 scrambled baseline trials. For AM in year 1 there were also 25 public event trials. The order of conditions was randomised, with the constraint that there were similar numbers of trials of each condition in every run.

During each trial, the participant saw a photograph which remained on the screen for 20 s. Participants were trained to follow these instructions: “Look at the picture, if you think it shows an event from your life, try and recollect the event being shown and try and re-live the experience. If you think it's not from your life [i.e. a foil], try and imagine what might have been going on.” [For AM's year 1 public events condition, the instruction was “…If you think it's not from your life, but that it's a famous public event, try and remember what happened during that event”.] For the scrambled pictures the instruction was to just look at it, try to relax and ‘empty your mind’.

After 20 s, the photograph was replaced by a question ‘*Remember? Y or N*’. The participant had to indicate using a key pad whether in general terms he remembered the event shown in the preceding photograph. Once he responded, or after 5 s has elapsed, the next stimulus appeared after a 500 ms gap. Because the participant had been trained extensively before scanning, he knew that he had up to 5 s to respond to each stimulus. Thus, the responses were self-paced. For the scrambled pictures the question was ‘*Ready? Y or N*’ (participants were trained to respond arbitrarily to this). The key pad responses were included to ensure attention was maintained during the experiment.

Overall, the paradigm was modelled on that of Gilboa et al. (2004), with two principal adjustments to the design. First, pilot work with AM and several elderly control participants indicated that 20″ was the optimal length of time to ‘re-experience’ a memory in response to a photographic stimulus. Any longer (e.g. the 30″ used by Gilboa et al., 2004) was too long, and prompted attention to waver. Second, instead of blocking memories of a similar age together in separate runs, we elected to randomise the presentation of memory ages and model the effects parametrically, thus eschewing the need for a categorical (and arbitrary) division between recent and remote memories.

After a scanning session and outside of the scanner, the participant was debriefed. The 25 autobiographical and the 25 foil photographs (and for AM in year 1, the public event photographs) were presented again (in the same order as they appeared during scanning) and the participant was required to say if they were from his life or not (to assess for false positive responses). This was also an opportunity for the participant to clarify his responses (i.e. to indicate if he pressed the wrong key by mistake in the scanner). If he thought a photograph was from his life, he had to describe what he had been thinking about each photograph during the scan, to indicate who was in the picture, what was happening, when it was, where it was, and whether he truly recollected the event or not. In AM's case, given his semantic dementia and its effect on his language, he did this by whatever means he could, through language, gestures, facial expressions, and noises.

Two examiners (EAM, MDK) were present for all of AM's post-scan debriefings. The examiners made notes independently during debriefing. The inter-rater reliability was 0.9. The final coding of post-scan ratings, in particular for those instances where the two examined differed, was achieved through collaboration at the end of each visit (in a similar manner to Westmacott et al. (2001) and Piolino et al. (2003)). As our interest was in the quality of AM's recollection and its brain basis, these in-depth ratings were used as the main behavioural measure. The examiners rated whether AM had recollected an event on the following scale:

0 = no memory at all for the contents of the photograph or the event
0.5 = one basic detail given such as people present or place, no memory of the event
1 = several details given, such as people and place, no memory of the event
1.5 = more details relevant to the event, no definite recollection of the event
2 = numerous details and a vague recollection of the event
2.5 = full details (who/what, when, where) and reasonable memory of the event
3 = full details and definite full sense of re-experiencing the event

Video recordings were made of each debriefing session. To assess the consistency of ratings over time, one of the examiners (EAM) viewed each year's video again (now several years since the original recordings were made), and blindly re-scored all of the memories. The correlation between the original scores and the new scores was 0.99, indicating consistency over time.

### 2.5 Neuroimaging data acquisition and analyses

T2 weighted echo planar (EPI) images with blood oxygen level dependent (BOLD) contrast were acquired on a 1.5 T Siemens Sonata MRI scanner. The following scanning parameters were used to achieve whole brain coverage: 36 slices, 2.5 mm thickness (1 mm gap), TR 3.24 s, TE 50 ms, field of view 192 mm, 64 × 64 matrix; in-plane resolution 3 mm × 3 mm, voxel size of 3 mm × 3 mm× 2.5 mm. The first five volumes from each run were discarded to allow for T1 equilibration effects.

Initially the data from AM's three visits were analysed separately using the statistical parametric mapping software SPM2 (http://www.fil.ion.ucl.ac.uk/SPM). In the first instance we sought to determine the possible effect of the normalization procedure on AM's brain, given the presence of atrophy. Thus, the images for each year were realigned but not normalized, and then co-registered to the relevant structural scan from that year. Having established the results for each year without the normalization procedure, we then analysed the data in the usual manner using SPM2, namely with spatial preprocessing consisting of realignment and normalization to a standard EPI template in MNI space with a resampled voxel size of 3 mm × 3 mm × 3 mm. The results with or without normalization were very similar for each year, thus we proceeded with a standard analysis using normalization, and smoothing using a Gaussian kernel with full width at half maximum of 8 mm, which allowed us to examine (and formally compare) the data from AM's separate visits within one model.

Following preprocessing, statistical analysis was performed using the general linear model (GLM). Our interest was in the 20 s period during which the participant was looking at the photographic stimuli. This period was modelled with a boxcar function (of 20 s duration) and convolved with the canonical haemodynamic response function to create regressors of interest. AM failed to recognize only 4/75 of his autobiographical memory photographs across the three years. Excluding such a small number of trials would make little difference to the overall results, therefore the data reported include all 75 autobiographical memory trials. Thus, the design matrix included regressors coding for each of the three main experimental conditions for each visit, autobiographical memory (*M*), foil photographs (*F*), control scrambled pictures (*C*) (and for AM at year 1, also public events). Two other regressors were also modelled for each autobiographical memory for each visit, the year the event occurred, and the examiners’ rating of the quality of AM's recollection. In addition, movement parameters for each visit were included as regressors of no interest. Condition-specific experimental effects (parameter estimates or regression coefficients) were obtained via the GLM in a voxel-wise manner. Visit-specific linear contrasts of these parameter estimates, collapsed across the three (four for year 1) runs of a visit, were entered into a series of one-sample *t* tests.

Four types of analysis were undertaken within this model: (1) We compared conditions within one visit (e.g. *M*_year1_ − *C*_year1_). (2) We also examined differences in activation patterns between visits. This was achieved by comparing an active (memory or foil) condition with the control condition and then comparing the result with the same contrast from another time point, e.g. (*M*_year1_ − *C*_year1_) − (*M*_year2_ − *C*_year2_). In this way, non-specific effects (e.g. scanner drift over time) were controlled as these would have affected the control and main tasks similarly at each time point. (3) As well as differences across time points, we were also interested in commonalities in activation patterns across visits, and examined this using conjunction analyses. (4) Finally, for each visit, we tested for any parametric changes in activity related to either memory age, or quality of recollection. The data from the control participant were preprocessed and analysed separately in the standard manner as described above. *p* < 0.005 was considered the criterion for significance in areas previously associated with autobiographical memory (see Gilboa et al., 2004; Hassabis, Kumaran, & Maguire, 2007; Maguire, 2001; Maguire & Frith, 2003; Maguire et al., 2001; Summerfield, Hassabis, & Maguire, 2009; Svoboda et al., 2006), but we report all activations at *p* < 0.005 for completeness. Areas outside the hypothesised autobiographical memory network were only considered significant if they survived correction at a threshold of *p* < 0.05 corrected. No areas survived correction at this threshold.

A T1-weighted structural MRI scan was also acquired during each visit for AM, and also for each of the ten control participants: 176 sagittal partitions were acquired with an image matrix of 256 × 224 (Read × Phase). Two-fold oversampling was performed in the read direction (head/foot direction) to prevent aliasing. The isotropic spatial resolution was 1 mm. Relevant imaging parameters were TR/TE/TI = 14.59 ms/4.15 ms/650 ms, BW = 85 Hz/Px, *α* = 20°. To increase the signal-to-noise ratio, an asymmetric position of the inversion pulse within the magnetisation preparation experiment (duration TI) was chosen, and the delay between the initial saturation and the inversion amounted to 40% of TI (Deichmann, Schwarzbauer, & Turner, 2004). The total duration of the scan was 12 min. Special RF excitation pulses were used to compensate for B1 inhomogeneities of the transmit coil (Deichmann, Good, & Turner, 2002). Images were reconstructed by performing a standard 3D Fourier Transform, followed by modulus calculation. No data filtering was applied either in *k* space or in the image domain.

Structural MRI images were analysed using the optimised VBM procedure implemented in SPM8. Briefly this involves a number of fully automated preprocessing steps including extraction of brain, spatial normalization into stereotactic (MNI) space, segmentation into grey and white matter and CSF compartments, correction for volume changes induced by spatial normalization (modulation), and smoothing with a 12 mm full width at half maximum (FWHM) isotropic Gaussian kernel. This level of smoothing is recommended in unbalanced designs such as this (Salmond et al., 2002). The preprocessing procedures used in SPM8 (and SPM5) have been shown to produce good results when matching brains with lesions to standardised templates (Crinion et al., 2007). Analyses focussed on grey matter. For each year separately, the patient's structural scan for that year and control scans were compared using a two sample *t*-test to investigate differences in grey matter volume. Given a priori interest in the temporal lobes, the significance level employed for these regions was *p* < 0.001 uncorrected for multiple comparisons. For completeness, we report all findings throughout the brain that survived correction at this threshold (Table S1 in Supplementary Materials). The significance level for the rest of the brain was set at *p* < 0.05 corrected for multiple comparisons.

## 3 Results

### 3.1 Behavioural data

#### 3.1.1 During scanning

The primary aim of the ‘Remember Y/N’ questions during scanning was to obtain a general indication of the participant's ability to distinguish his memories from the highly similar foil photographs. It was also a means of checking if the participant maintained attention during the experiment. The scores on Table 2 show that on each occasion AM was able to distinguish his own autobiographical memories from the foils effectively, and in a similar manner to the control participant. This was in marked contrast to his ability to identify and recall public events, where he recalled less than half.

Although the responses during scanning were not reaction times, on each occasion AM took on average less than one second to respond to autobiographical stimuli, similar to the control participant (see Table 2). His responses were also comparable and consistent across conditions and visits.

#### 3.1.2 Post-scan

The main focus of this study was on the quality of AM's autobiographical recollection and how it might be affected by disease progression. Recollection quality was measured by experimenter ratings that were made post-scan during the debriefing session.

**Fig. 2:**
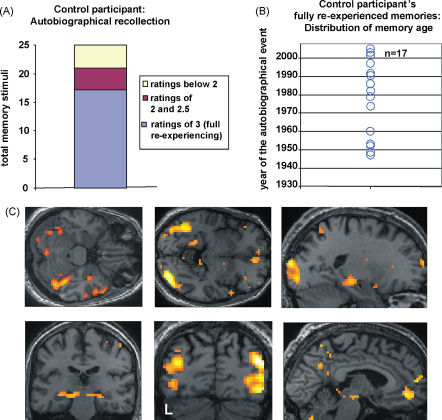
Control participant: recollection of autobiographical memories. (A) The bar in this graph represents the total number (25) of autobiographical memory photograph stimuli used in the scanning experiment. The legend beside the graph indicates the share of the rating types. See also Table 2. (B) A plot showing those memories that were fully recollected and re-experienced by the control participant, with the year the event happened shown on the *y*-axis. *N* = 17 for fully re-experienced memories – note that some data points overlap C. The brain network supporting retrieval of autobiographical memories in the control participant. See Table 3 for full details of activation locations. Views of this distributed brain network are shown (*p* < 0.005 uncorrected) on a selection of relevant axial, sagittal and coronal sections from the control participant's structural MRI. L: left side of the brain.

**Fig. 3:**
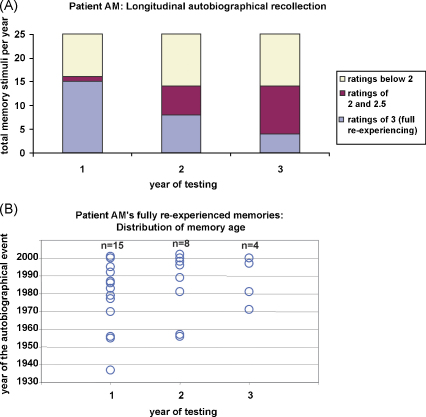
Profile of patient AM's autobiographical recollection over time. (A) The bars in this graph represent the total number (25 each year) of autobiographical memory photograph stimuli used in the scanning experiment for each of the three years. The stimuli were different on every occasion. The legend beside the graph indicates the share of the rating types for each visit. See also Table 2. (B) Plots showing those memories that were fully recollected and re-experienced by the control participant each year of testing, with the year the event happened shown on the *y*-axis. Note that for year 1, some data points overlap.

Considering firstly the control participant, he had a mean rating of 2.48 (Table 2). Overall, he scored the top ‘full vivid re-experiencing’ rating (of 3) for 17/25 memories (Fig. 2A). AM's year 1 mean rating for autobiographical memories of 2.16 was similar, and15/25 were ‘fully re-experienced’ (see Fig. 3A). A chi-square test was used to compare AM (at year 1) and the control participant in terms of the frequency of each rating score for recollection. There was no significant difference between the patient and control participant in the frequency of the ratings (*χ*^2^ = 6.79, d.f. = 6, *p* = 0.34). Thus, at year 1 of the study, the quality of AM's recollection was similar to a healthy control participant where in both cases the stimuli had been pre-selected by their wives, without their involvement. Of note and in line with his in-scanner score, his mean public event rating for his first visit was less than 1 (see Table 2), and he achieved a ‘fully re-experienced’ rating for only 2/25 stimuli. This indicated that AM had an impairment in his memory of public events which might be related to his general deficit in semantic knowledge.

Over the next two visits, AM's mean rating for autobiographical memory declined (see Table 2). This was primarily due to a shift from full re-experiencing ratings of 3 to ratings of 2.5 and 2, whilst the frequency of lower ratings remained constant (see Fig. 3A). There was a significant effect of disease progression (over the course of the three years) on ratings of recollection (*χ*^2^ = 24.16, d.f. = 12, *p* = 0.025).

What is notable from Fig. 3B is that there appears to be no effect of the recency/remoteness of the autobiographical memories. That is, those memories that achieved a full re-experiencing rating were distributed across the decades (as were those of the control participant – Fig. 2B), and were not just memories for events that had occurred more recently. Even as AM's number of fully re-experienced memories declined over time, neither primacy nor recency effects were apparent (i.e. no temporal gradient or reversed gradient). In addition, a correlation analyses failed to show any significant relationship between the age of a memory and ratings of recollective quality (year 1: *r* = 0.27, *p* = 0.19; year 2: *r* = 0.20, *p* = 0.33; year 3: *r* = 0.08, *p* = 0.68; control participant *r* = 0.21, *p* = 0.32). We formally examined the distribution of memories in two ways, first by using a Kolmogorov–Smirnov (K–S) test to ascertain if the fully recollected memories at year 1 for AM and the control participant were taken from same distribution. We found that they were (*D* = 0.24; *p* = 0.71). We also established using K–S tests that for both AM (*p* = 0.49) and the control participant (*p* = 0.55) the data were consistent with a normal distribution. This was also the case for AM's fully recollected memories at year 2 (*p* = 0.76) and year 3 (*p* = 0.88). These results accord with the view that in this case, there was no evidence of a temporal gradient.

### 3.2 fMRI data

#### 3.2.1 Control participant

In the first instance, we verified that the task used gave rise to the expected brain network in the control participant, a network that has been found repeatedly in fMRI studies of autobiographical memory (see Gilboa et al., 2004; Hassabis, Kumaran, & Maguire, 2007; Maguire, 2001; Maguire & Frith, 2003; Maguire et al., 2001; Summerfield et al., 2009; Svoboda et al., 2006). The contrast of autobiographical memory recall with the low level control task indeed revealed the ‘classic’ autobiographical memory network in the control participant (see Fig. 2C, Table 3), which included medial prefrontal cortex, anterior and lateral temporal cortex, medial temporal areas including both hippocampi, retrosplenial cortex, and medial parietal areas. As the stimuli in the control task lacked any discernible objects/features, there was also increased activation in posterior temporal and occipital areas for the autobiographical memory photographs. Having verified that the task produced the expected brain responses in a healthy control participant, the main analyses focused on patient AM.

#### 3.2.2 Patient AM

##### 3.2.2.1 Within-visit comparisons and commonalities

**Fig. 4:**
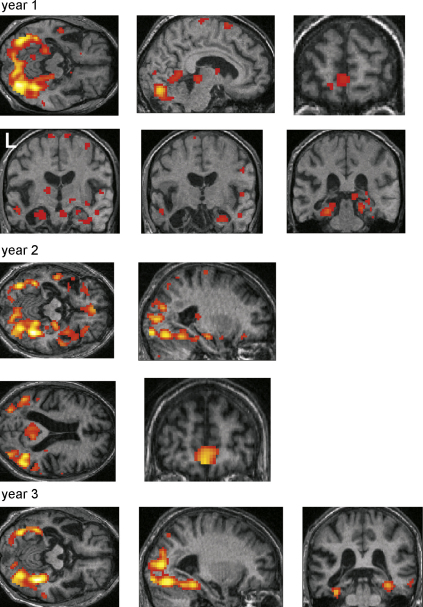
The brain networks associated with autobiographical memory retrieval in patient AM. Brain areas more active during autobiographical memory retrieval compared with the control task are shown for each year. Functional images are shown on a selection of relevant axial, sagittal and coronal sections from the patient's structural MRI scan contemporaneous with the functional images for that year. See Table 4 for full details of activation locations. Activations are shown at a threshold of *p* < 0.005 uncorrected. L: left side of the brain.

We first ascertained the brain areas that were active during the recall of autobiographical events compared with the low level control task for each year AM was tested. Full details of the results are reported in Fig. 4 and Table 4. To summarise, at year 1 and year 2 the ‘classic’ autobiographical brain network was activated including medial prefrontal cortex, anterior and lateral temporal cortex, medial temporal areas including hippocampus, retrosplenial cortex, and medial parietal areas. As with the control participant, because the stimuli in the control task lacked any discernible objects/features, there was also increased activation in posterior temporal and occipital areas for the autobiographical memory photographs. Given the extent of his left temporal neocortical atrophy it is interesting to note that the residual tissue was still active. This was also true at year 1 for both hippocampi, and the right hippocampus at year 2.

**Fig. 5:**
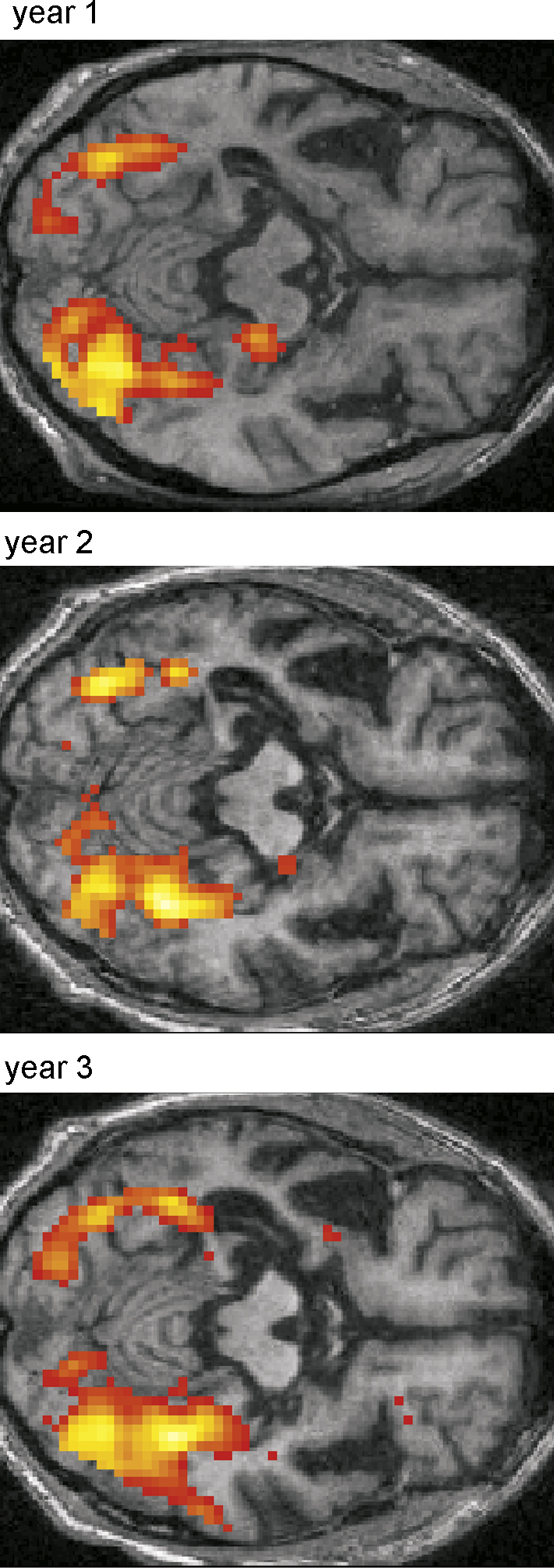
The brain areas associated with processing foil photographs. Brain areas more active during processing of foil photographs compared with the control task are shown for each year. Functional images are shown on an axial section from the patient's structural MRI scan contemporaneous with the functional images for that year. Activations are shown at a threshold of *p* < 0.005 uncorrected. For year 1, brain areas activated were: right hippocampus (36, −33, −12; *Z* = 3.35); left posterior parahippocampal gyrus/fusiform cortex (−36, −42, −27; *Z* = 5.16); left lateral occipital cortex/posterior fusiform cortex (−42, −54, −18; *Z* = 5.65); right lateral occipital cortex/posterior fusiform cortex (36, −54, −21; *Z* > 8); right, superior part of middle occipital gyrus (33, −84, 9; *Z* > 8); right middle occipital gyrus (45, −81, −9; *Z* > 8); left inferior occipital gyrus (−36, −78, −21; *Z* > 8). Year 2: right hippocampus/parahippocampal gyrus (35, −36, −16; *Z* = 4.0); left posterior parahippocampal gyrus/fusiform cortex (−36, −42, −27; *Z* = 5.71); right fusiform cortex (36, −54, −21; *Z* > 8); left lateral occipital cortex/posterior fusiform cortex (−42, −48, −21; *Z* = 5.61); right lateral occipital cortex/posterior fusiform cortex (36, −54, −21; *Z* > 8); right, superior part of middle occipital gyrus (33, −84, 6; *Z* = 5.83); right middle occipital gyrus (45, −78, −9; *Z* > 8); left inferior occipital gyrus (−36, −75, −21; *Z* = 7.62). Year 3: left posterior parahippocampal gyrus/fusiform cortex (−36, −42, −27; *Z* = 7.64); right posterior parahippocampal gyrus/fusiform cortex (29, −35, −26; *Z* = 5.0); right fusiform cortex (33, −48, −18; *Z* = 7.1); left lateral occipital cortex/posterior fusiform cortex (−42, −51, −18; *Z* = 6.36); right lateral occipital cortex/posterior fusiform cortex (36, −51, −18; *Z* > 8); right, superior part of middle occipital gyrus (30, −84, 6; *Z* = 7.76); right middle occipital gyrus (45, −81, −9; *Z* > 8); left inferior occipital gyrus (−36, −78, −21; *Z* = 6.24). Cross-year comparisons revealed only one significant result: foil-control task year 1 > foil-control task year 3, right hippocampus (21, −27, −15; *Z* = 4.80).

A notable change had occurred by year 3. On this final occasion there were many fewer brain areas activated in response to autobiographical memory photographs, with posterior occipito-temporal activity predominating. Interestingly, the pattern of activity for autobiographical memory in year 3 bears a striking resemblance the patterns of brain responses elicited by the unfamiliar foil photographs (when compared with the control task) on each year of testing — see Fig. 5. The public events photographs, many of which AM did not recognise at year 1, also elicited the same pattern (see Fig. S1 in Supplementary Materials).

Using conjunction analyses it was possible to examine more formally the brain areas that were activated in common across the years in response to autobiographical memory (see Fig. S2 in Supplementary Materials). We first asked what was active in common for years 1 and 2. Unsurprisingly, many of the areas detailed on Table 4 for these two years were found to be active in common. However, when we asked what areas were active in common across the three years, again it is not surprising that the only areas in common were posterior occipito-temporal regions. This reflects the reduced extent of activation in year 3 of the experiment for autobiographical memory.

##### 3.2.2.2 Between-visit comparisons

**Fig. 6:**
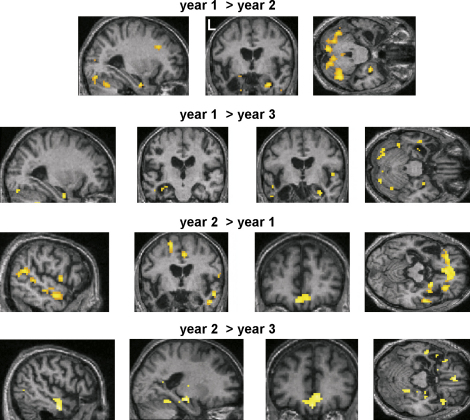
Direct comparisons across years for autobiographical memory. Functional images are shown on a selection of relevant sagittal, coronal and axial sections from the mean structural MRI scan of the patient averaged across the three years. See Table 5 for full details of exact contrasts and activation locations. Activations are shown at a threshold of *p* < 0.005 uncorrected. L: left side of the brain.

After ascertaining the brain networks active in response to autobiographical memory retrieval for each year separately and establishing the areas that activated in common across the three years of the study, we next sought to examine differences between visits. The results relating to autobiographical memory are reported in detail in Fig. 6 and Table 5.

Three differences are particularly notable. First, the change that seemed apparent from the within-visit comparisons and conjunction analyses by year 3 was confirmed, with significantly greater activity across the memory network in years 1 and 2 compared with year 3.

The second finding of note relates to the status of the hippocampi. Although already compromised structurally at year 1, the left hippocampus was nevertheless active in response to autobiographical stimuli, along with the more intact right hippocampus. Although at year 2 the main contrast (Table 4) showed right hippocampus and now no left hippocampal activation, direct comparison of year 1 and year 2 showed no differences in left hippocampus suggesting that even at year 2 in a very compromised and reduced state, it was still active, perhaps at a sub-threshold level. Interestingly, there was greater activity in the right hippocampus at year 1 than year 2; this is perhaps an indication of the spread of the atrophy, including increased left hippocampal atrophy as well as the beginnings of atrophy in the right medial temporal lobe.

Whilst there were no brain areas more active at year three compared with any other year, the same was not true for year 2. Interestingly, during autobiographical memory retrieval there was significantly more activity in ventromedial and ventrolateral prefrontal cortices compared with either year 1 or year 3. In addition, activation was increased in right temporal neocortex and in left and right precuneus. Thus, it would appear that whilst the classic autobiographical memory brain system is in operation at year 1, at year 2 this system is being up-regulated in frontal, temporal and medial parietal areas of the network. This may reflect a memory system under stress from the progressive pathology, and its attempt to maintain ‘business as usual’. However, by year 3 of the study, the situation has evolved further, and activity overall is greatly reduced. Interestingly this up-regulation in year 2 was not a general effect, but was specific to autobiographical memory retrieval. There were no differences in these frontal, temporal and parietal areas when foil conditions were compared across years [e.g. (*F*_year2_ − *C*_year2_) − (*F*_year1_ − *C*_year1_)] (see Fig. 5).

##### 3.2.2.3 Additional analyses

We also assessed whether any brain areas in AM showed changes in activity in response to the age of autobiographical memories. No such changes were apparent for any of the visits. Similarly, there were no changes in activity associated with the ratings of recollective quality (this was also the case for the control participant). Given that the data relate to a single participant, these data should be interpreted with caution.

Whilst our main interest was in examining AM's autobiographical memory network and this was best achieved by comparison with the low level control task, for each visit we also compared directly the autobiographical and foil conditions (see Fig. S3 in Supplementary Materials). Brain areas that distinguished real memories from the very similar foils included posterior cingulate cortex, angular gyrus, precuneus, and medial prefrontal cortex at year 1 and also year 2 (similar areas were active in the control participant for this contrast – see legend of Fig. S3). No differences in brain activity were apparent between the autobiographical memory and foil conditions at year 3, and no areas were more active for foils than autobiographical memories for any of the visits.

Whilst we report greater activation in a number of regions for the contrast of autobiographical memory compared with the control scrambled stimuli, it is possible that activity for the memory condition remained constant and there was a reduction of activity in the control condition between years. However, we found no significant differences between any of control conditions across the three years (i.e. *C*_year1_ vs *C*_year2_ and vice versa; *C*_year2_ vs *C*_year3_ and vice versa; *C*_year1_ vs *C*_year3_ and vice versa). This is further evidence that the observed changes are specific to autobiographical memory processing.

## 4 Discussion

This study adds to the relatively small literature on functional neuroimaging of memory in patients with damage or severe atrophy within critical brain circuitry (Chakraborty & McEvoy, 2008; Cipolotti & Maguire, 2003; Hassabis, Kumaran, Vann, & Maguire, 2007; Iaria, Bogod, Fox, & Barton, 2009; Maguire, Frith, Rudge, & Cipolotti, 2005; Maguire et al., 2001; Rosenbaum, Winocur, Grady, Ziegler, & Moscovitch, 2007). Importantly it is, to our knowledge, the first study to investigate change through time of memory-related fMRI activity in a patient with progressive pathology. We found that patient AM's autobiographical memories were relatively robust in the face of advancing atrophy. Furthermore, despite significant volume loss, his residual hippocampal and temporal neocortical tissue were active during recollection. Our results also revealed possible compensatory mechanisms that underpinned his autobiographical recall in the face of increasing structural compromise of his memory system. These involved ventromedial and ventrolateral prefrontal cortex, right temporal neocortex, and left and right precuneus. We did not find evidence of a reversed temporal gradient either behaviourally or in the fMRI data in this case. We first consider the nature of AM's autobiographical recollection, and then consider its neural basis.

We found that in spite of very compromised semantic memory and language abilities, initially, AM's recollection of autobiographical events was preserved, and he scored a similar number of ‘3’ ratings (full re-experiencing of an event) as the control participant at year 1. Over subsequent years, there was a gradual decrease in the quality of recollection, with a shift to ratings of ‘2.5’ and ‘2’, whilst ratings below ‘2’ (indexing recall of semantic information but no episodic recall) stayed consistent across years. Thus AM's autobiographical memory retrieval was not devastated at any time during this study, despite his left hippocampus and temporal neocortex being quite severely atrophied initially and then increasing atrophy which also involved the right side. Rather, the progressive pathology resulted in a gradual erosion of the full recollective experience, but still left AM able to recognise photographs depicting events from his own life, able to recall details about them, and to retain, even at year 3, some ability to re-live the event. This pattern stands in marked contrast to patients with Alzheimer's disease (Becker & Overman, 2005; Kopelman, 1989) and also some patients with selective bilateral hippocampal pathology, where damage can compromise recollection of autobiographical events to a much greater degree (Rosenbaum et al., 2008, but see Bright et al., 2006; Squire & Bayley, 2007).

It could be argued that the pattern of memory preservation in AM may reflect a reverse temporal gradient, i.e. preserved recall of recent autobiographical events but compromised recall of remote events. Such a pattern has been reported in several studies (Graham & Hodges, 1997; Graham et al., 2003; Hou et al., 2005; Nestor et al., 2002; Piolino et al., 2003; Snowden et al., 1996), although others have failed to replicate this finding (McKinnon et al., 2006; Moss et al., 2003; Westmacott et al., 2001). We found no evidence for a temporal gradient in AM's recall (or indeed any modulation of brain activity in response to memory remoteness). In the first year, his autobiographical memory recall was intact, and he recollected recent memories and those from many decades ago equally well. Even when the quality of his recollection started to wane, those memories that were fully recollected were from both recent and remote time periods. Reasons for discrepancies in the literature concerning temporal gradient of recall in SD may be due to the numbers of memories tested, the way memories are cued, the severity and location of brain atrophy, and the difficulty of assessing autobiographical memory in patients with significant semantic and language impairments. Our study of patient AM included testing the recall of 75 unique autobiographical events that spanned eight decades, and two raters who used a detailed 7 point scale and took into account both verbal and non-verbal communications from AM. Whilst our findings in themselves do not allow us to differentiate between the standard consolidation model of memory (Squire, 1992) and other theories that emphasise the role of the hippocampus in autobiographical memory retrieval in perpetuity (Hassabis & Maguire, 2009; Nadel & Moscovitch, 1997), the continued activation of AM's hippocampi during retrieval of remote autobiographical memories is more consistent with predictions of the latter models.

How are the patterns of autobiographical memory retrieval in AM sustained at the neural level? In healthy participants, recollection of personal past experiences activates a well-established and distributed network of brain areas, including the hippocampi (particularly on the left), other medial temporal lobe areas, anterior and lateral temporal cortex, medial prefrontal cortex, retrosplenial cortex, and precuneus (Maguire, 2001; Svoboda et al., 2006). Our task, adapted from Gilboa et al. (2004), resulted in activation of this classic network in the control participant. Our question was what would happen to activations in this network in the presence of progressive pathology, and relatively intact autobiographical memory recall. We reasoned that there may be a number of different consequences, for example, residual tissue in atrophied regions might still activate, there could be up-regulation of areas within the network, or there could be recruitment of additional brain areas into the network. In fact, all three phenomena occurred.

AM's intact autobiographical recollection at year 1 was accompanied by activation of the classic memory network. In particular, the residual tissue in his left hippocampus and left lateral neocortex were engaged during recall. Thus, even when there is significant volume loss in key brain areas such as the hippocampus, this does not mean that the residual tissue is not active or potentially making a contribution to recall. This may be one factor explaining why there are discrepancies in the literature between patients who have limited retrograde amnesia (Squire & Bayley, 2007) and others who have retrograde amnesia extending for many decades (Moscovitch et al., 2005; Nadel & Moscovitch, 1997; Rosenbaum et al., 2008). We suggest that volume of the hippocampus alone cannot be used to infer function (Bright et al., 2006; Kopelman et al., 2003). As the current case illustrates, AM's significantly atrophied left hippocampus was active and may have contributed to his preserved autobiographical memory. However, in another patient, the remnant tissue might not be viable, and so with the same degree of volume loss, there would be a very different pattern of autobiographical memory recall (Hassabis, Kumaran, Vann, et al., 2007; Maguire et al., 2001, 2005). The use of fMRI in patients with hippocampal damage is still rare but perhaps should be undertaken more often to help to characterise the functionality of residual tissue.

At year 2, the usual memory retrieval network was active, with the exception of the left hippocampus, atrophy of which now compromised its engagement. AM's autobiographical recollection had decreased slightly in quality but he was nevertheless still able to recall and re-live events. This seems to have been achieved by the operation of two mechanisms. First, activity in several areas within the memory retrieval network was up-regulated, namely ventromedial prefrontal cortex (vmpfc), right lateral temporal cortex (in particular around the superior temporal sulcus) and the precuneus. This up-regulation was specific to autobiographical memory, as no such effects were associated with the foil stimuli at year 2. Neither is this up-regulation a feature of aging in general. Maguire and Frith (2003) compared healthy elderly (average age 75) with younger (average age 32) participants on autobiographical memory retrieval during fMRI and found greater engagement of the hippocampus bilaterally in the elderly, but no differences in other brain regions.

In the case of AM, we speculate that the vmpfc may be compensating to some degree for the hippocampus. Vmpfc has reciprocal connections with the medial temporal lobe, and widespread connections to the neocortex. It has been suggested this region, given its connectivity, may play a role in integrating information from multiple neocortical regions during memory retrieval (Takashima et al., 2006). It is possible that at year 2 its integrative role expands to assume duties previously performed by the hippocampus. It may be assisted in this endeavour by the precuneus, which has been implicated in studies of recognition memory, with increased activity in response to familiar items (Hornberger, Rugg, & Henson, 2006; Rugg, Otten, & Henson, 2002; Vincent et al., 2006; Wagner, Shannon, Kahn, & Buckner, 2005). Hassabis, Kumaran and Maguire (2007) also showed that real autobiographical memories engaged the precuneus (and vmpfc) more than imagined fictitious experiences. This was also the case for patient AM at years 1 and 2, with real memories engaging these areas to a greater extent than the foil photographs. The greater precuneus activity at year 2 may reflect an increased dependence on using familiarity cues to support recollection. The increased activation in lateral temporal regions at year 2 may reflect the decline in AMs semantic memory, with increasing effort required to process the semantic content of the stimuli. However, it should also be noted that lesion studies have previously implicated right temporal and frontal areas in autobiographical memory retrieval (Bright et al., 2006; Buccione et al., 2008; Kopelman et al., 1999; Markowitsch et al., 1993; O’Connor et al., 1992; Ogden, 1993).

Ventrolateral prefrontal cortex (vlpfc) is often reported to be engaged during autobiographical memory retrieval (Svoboda et al., 2006) although not invariably. Its recruitment may reflect the nature of the stimuli and the manner in which autobiographical memory recall is tested. This region was not activated in the Gilboa et al. (2004) study on which our paradigm was based. That vlpfc was not activated by AM or the control participant at year 1 may therefore reflect the reduced need for this region when rich photographic stimuli are employed. However, this area was engaged in AM at year 2, and may signal another compensatory mechanism underpinning the attempt to keep the autobiographical memory retrieval system operational. It has been suggested this region contributes to autobiographical memory retrieval by engaging in strategic retrieval, verification, and selection of information from posterior cortical association areas (see Svoboda et al., 2006). Photographic stimuli may normally eschew the need for some of these processes, however, in the context of encroaching pathology, they may become necessary in order to boost recollection.

By year 3, a different pattern of brain activations had emerged. Whilst AM was still able to discriminate between his own and foil stimuli, the majority of recollections were now rated between ‘2’ and ‘2.5’ with only a small number given a full ‘3’ rating. There was disengagement of numerous brain areas, and compensatory mechanisms did not appear to be operating. Four months after this final fMRI scan, further behavioural testing of AM, now in an advanced stage of SD, revealed that his autobiographical memory retrieval had collapsed (see Table 1). Whilst he was tested on this occasion using the Autobiographical Memory Interview (Kopelman et al., 1990), a verbal task which may have disadvantaged him, AM's wife also confirmed a marked deterioration in his autobiographical memory. Thus it would seem that the behavioural and fMRI findings at year 3 were signalling the imminent collapse of his memory system.

Whilst AM appears to be a typical case of semantic dementia (or progressive fluent aphasia), secondary to focal temporal lobe atrophy, as in any report of a single patient, there are caveats. It could be argued that such patients offer limited statistical power. In addition, the patterns of functional deficits may differ from patient to patient (as might the degree of hippocampal atrophy) and we acknowledge that SD can vary in its presentation. Therefore, further patients need to be examined using fMRI in order to test the generalisability of our findings.

In summary, the existence of an unusually large set of photographic stimuli allowed us to examine AM's autobiographical memory in a very detailed fashion and longitudinally through the course of his disease both behaviourally and using fMRI. He also remained sufficiently cooperative to be scanned for the duration of the study. This enabled us to plot the course of autobiographical memory during SD: we established that there was no clear-cut evidence of a temporal gradient in this case, but rather a gradual loss in recollective quality that collapsed only late in the course of the disease. Moreover, we have shown that atrophied tissue can still activate and in an appropriate fashion, and that this is subsequently augmented by compensatory up-regulation of other parts of the memory system including frontal and right temporal cortices. A further step-change in the brain areas engaged and in the quality of recollection then preceded a collapse of our patient's autobiographical memory. These findings inform key theoretical debates about the role of the hippocampus and neocortical areas in supporting remote autobiographical memories. Moreover, our results suggest it may be possible to define specific stages in SD-related memory decline, and that fMRI (perhaps using a simplified version of our protocol) could complement MRI and neuropsychological measures in providing more precise prognostic and rehabilitative information for clinicians and carers.

## Figures and Tables

**Table 1:** AM's background neuropsychological test scores.

Measure	November 2001	January/February 2002	October 2003	August/October 2004	October 2005 (4 months post-study)
WASI-FSIQ	109				
Modified Card-sorting Test					
-Categories	5/6				
-% Perseverations	13				

Graded Naming test	4/30^a^				0/30^a^
Semantic fluency (six categories)					
-Living	24				
-Non-living	19				
FAS verbal fluency test	27 (25th percentile)				
British Picture Vocabulary Scale		110/150^a^		56/150^a^	
Snodgrass naming test	97/120				

Reading					
-Regular	37/39	39/39	38/39	35/39	
-Irregular	37/39	35/39	32/39^a^	27/39^a^	
-Non-words	56/60				
-NART–irregular (/50)	30 errors				

Pyramid & Palm Trees Test					
-Words	49/52				
-Pictures	47/52			47/52	44/52^a^
PALPA word-picture matching		39/40	35/40	32/40^a^	

WMS-R					
-Logical memory					
-Immediate recall	6/50 (3rd percentile)^a^				
-Delayed recall	4/50 (10th percentile)^a^				
-Visual reproduction					
-Immediate recall	29/41 (72nd percentile)				
-Delayed recall	20/41 (66th percentile)				

Recognition Memory Test					
-Words	41/50 (38th percentile)				
-Faces	37/50 (7th percentile)^a^				

Autobiographical Memory Interview					
-Personal semantics					
-Child	7/21^a^				2.5/21^a^
-Young adult	10/21^a^				0/21^a^
-Recent	15.5/21^a^				0.5/21^a^
-Autobiographical incidents					
-Child	8/9				1.5/9^a^
-Young adult	4/9^a^				0/9^a^
-Recent	8/9				0/9^a^

WASI: Wechsler Abbreviated Scale of Intelligence; NART: National Adult Reading Test; PALPA: Psycholinguistic Assessments of Language Processing in Aphasia; WMS-R: Wechsler Memory Scale Revised.

a Impaired.

**Table 2:** Behavioural data and ratings.

Measure	Patient	Control participant
‘Remember Y/N?’ questions in the scanner:		
Year 1		
Autobiographical memory	23/25; 0 false positives	23/25; 1 false positive^a^
Public event memory	10/25	–

Year 2		
Autobiographical memory	25/25; 0 false positives	–

Year 3		
Autobiographical memory	23/25; 4 false positives^b^	–

Mean (S.D.) response times (ms) in the scanner:		
Year 1		
Autobiographical memory	640.39 (321.5)	708.32 (98.91)
Public event memory	707.96 (242.79)	–
Foil photographs	794.08 (426.52)	346.27 (82.31)

Year 2		
Autobiographical memory	849.72 (287.94)	–
Foil photographs	940.24 (446.47)	–

Year 3		
Autobiographical memory	703.52 (211.61)	–
Foil photographs	722.17 (151.68)	–

Post-scan (experimenters’) ratings:^c^		
Mean (S.D.) rating [0–3]		
Year 1		
Autobiographical memory	2.16 (1.11)	2.48 (0.98)
Public event memory	0.96 (1.09)	–

Year 2		
Autobiographical memory	1.9 (0.9)	–

Year 3		
Autobiographical memory	1.66 (0.99)	–

Sum of ratings [/75]		
Year 1		
Autobiographical memory	54	62
Public event memory	24	–

Year 2		
Autobiographical memory	47.5	–

Year 3		
Autobiographical memory	41.5	–

a False positive response due to a mistaken key press.

b 2 of the false positive responses due to mistaken key presses.

c See also Figs. 2 and 3.

**Table 3:** Control participant: autobiographical memory > control task.

Region	Peak coordinate (*x*, *y*, *z*)	*Z*
Ventromedial prefrontal cortex	0, 51, −15	5.88
Anterior medial prefrontal cortex	3, 63, 9	5.36
Left temporal pole	−36, 21, −33	4.60
Right temporal pole	39, 21, −33	3.86
Right superior temporal sulcus	57, 3, −24	3.89
Left hippocampus	−30, −18, −18	4.72
Right hippocampus	24, −21, −18	4.86
Left posterior parahippocampal gyrus	−24, −39, −12	5.10
Left retrosplenial cortex	−3, −60, 9	4.67
Right posterior cingulate cortex	6, −63, 27	4.68
Left lateral occipital/posterior fusiform cortex	−42, −63, −15	6.74
Right lateral occipital/posterior fusiform cortex	42, −54, −18	7.23
Left, superior part of middle occipital gyrus	−39, −84, 18	7.67
Right, superior part of middle occipital gyrus	45, −81, 18	>8
Left, inferior part of middle occipital gyrus	−39, −87, −3	6.48
Right, inferior part of middle occipital gyrus	33, −87, −3	>8
Right posterior cerebellum	6, −75, −45	4.90

**Table 4:** Patient: autobiographical memory > control task.

Region	Peak coordinate (*x*, *y*, *z*)	*Z*
Year 1:		
Ventromedial prefrontal cortex	−3, 60, −3	4.13
Left temporal pole	−30, 6, −30	3.46
Left superior temporal sulcus	−54, −12, −18	4.51
Right superior temporal sulcus	51, −3, −27	4.83
Left hippocampus	−27, −15, −24	2.9
Right hippocampus	18, −27, −15	5.54
Left parahippocampal gyrus	−24, −36, −21	6.32
Right parahippocampal gyrus	21, −33, −18	5.07
Right retrosplenial cortex	6, −57, 3	6.11
Left posterior cingulate cortex	−12, −54, 33	4.27
Left lateral occipital/posterior fusiform cortex	−42, −51, −21	>8
Right lateral occipital/posterior fusiform cortex	36, −54, −21	>8
Right superior occipital gyrus	33, −78, 21	5.20
Left inferior occipital gyrus	−26, −78, −21	>8
Right inferior occipital gyrus	36, −75, −18	>8
Left cerebellum	−3, −78, −21	>8

Year 2:		
Ventromedial prefrontal cortex	0, 51, −15	6.37
Left lateral ventral prefrontal cortex	−30, 33, −18	3.81
Right lateral ventral prefrontal cortex	30, 33, 18	4.22
Left temporal pole	−30, 3, −21	4.02
Right temporal pole	45, 15, −21	3.85
Left superior temporal sulcus	−54, −18, −15	5.63
Right superior temporal sulcus	51, −3, −27	7.30
Right hippocampus	24, −18, −18	5.54
Left posterior parahippocampal gyrus/fusiform cortex	−33, −39, −27	7.60
Right parahippocampal gyrus	27, −33, −21	4.48
Right retrosplenial cortex	6, −51, 12	5.14
Posterior cingulate cortex	0, −48, 27	5.83
Precuneus	−3, −60, 45	>8
Left temporo-parietal junction	−48, −66, 18	5.78
Right temporo-parietal junction/horizontal posterior segment of superior temporal sulcus	51, −66, 12	>8
Left lateral occipital/posterior fusiform cortex	−39, −51, −15	4.14
Right lateral occipital/posterior fusiform cortex	33, −51, −18	7.0
Right middle occipital gyrus	45, −81, −9	>8
Left inferior occipital gyrus	−39, −75, −21	>8

Year 3:		
Left posterior parahippocampal gyrus/fusiform cortex	−36, −39, −27	6.66
Right fusiform cortex	36, −48, −18	>8
Left lateral occipital/posterior fusiform cortex	−42, −51, −18	5.84
Right, superior part of middle occipital gyrus	33, −84, 9	7.36
Right middle occipital gyrus	45, −81, −9	>8
Right inferior occipital gyrus	30, −75, −15	7.43
Left inferior occipital gyrus	−39, −75, −18	6.56

**Table 5:** Patient: autobiographical memory > control task, comparisons across years.

Region	Peak coordinate (*x*, *y*, *z*)	*Z*
Year 1 > Year 2:^a^		
Right dorsolateral prefrontal cortex	36, 27, 33	3.76
Right anterior hippocampus	30, −6, −33	3.79
Left inferior occipital gyrus	−42, −60, −24	3.35
Right inferior occipital gyrus	39, −66, −27	5.40
Left cerebellum	−9, −84, −33	3.72
Right cerebellum	42, −57, −42	4.59

Year 1 > Year 3:^b^		
Left hippocampus	−27, −21, −21	2.67
Right anterior hippocampus	30, −6, −30	3.25
Right hippocampus/entorhinal cortex	21, −27, −15	3.26
Medial inferior occipital cortex	−3, −81, −21	4.49
Left inferior occipital gyrus	−24, −78, −21	3.96
Left cerebellum	−39, −54, −45	3.24
Right cerebellum	39, −54, −42	4.14

Year 2 > Year 1:^c^		
Ventromedial prefrontal cortex	−3, 21, −12	5.19
	−6, 39, −21	4.17
Left lateral ventral prefrontal cortex	−30, 33, −18	3.97
Right lateral ventral prefrontal cortex	30, 30, −18	3.63
Right insula	24, 3, −18	4.25
Right superior temporal sulcus	48, −3, −33	4.56
Right superior temporal gyrus	63, −36, 3	4.09
Right middle temporal gyrus	60, −18, −12	4.09
Right temporo-parietal junction	39, −63, 15	4.02
Left precuneus	−6, −57, 45	4.36
Right precuneus	12, −48, 51	4.36

Year 2 > Year 3:^d^		
Ventromedial prefrontal cortex	6, 42, −9	3.89
Left lateral ventral prefrontal cortex	−33, 24, −12	3.43
Right hippocampus	24, −18, −18	3.74
Left inferior temporal sulcus	−51, −12, −30	3.68
Right superior temporal gyrus	63, −39, 3	3.70
Right anterior temporal cortex	42, 24, −33	3.30
Left precuneus	−9, −57, 42	3.81
Right precuneus	12, −51, 33	3.21

*Note*. No areas were more active for (memory-control task in year 3) compared with (memory-control task in year 1) or (memory-control task in year 2).

a Contrast [(memory-control task in year 1) > (memory-control task in year 2)].

b Contrast [(memory-control task in year 1) > (memory-control task in year 3)].

c Contrast [(memory-control task in year 2) > (memory-control task in year 1)].

d Contrast [(memory-control task in year 2) > (memory-control task in year 3)].
