# Supplementary material for: Autobiographical memory in semantic dementia: A longitudinal fMRI study
Source: Neuropsychologia. 2010 Jan;48(1):123–36. doi: 10.1016/j.neuropsychologia.2009.08.020 (PMC2806951; doi:10.1016/j.neuropsychologia.2009.08.020)
Supplement: Supplementary file 1 [file mmc1.doc]

**Autobiographical memory in semantic dementia: a longitudinal fMRI study**

**Maguire et al.**

***Supplementary Materials:***

**Table S1. VBM analysis of AM’s structural MRI brain scans**

Region Peak Coordinate (x, y, z) Z

*Areas of grey matter volume loss in AM vs. 10 matched control participants at Year 1*

Left superior temporal sulcus -42, 2, -16 5.60

Left superior temporal gyrus -54, -4, -14 5.02

Left body-posterior hippocampus -24, -24, -16 4.98

Left anterior hippocampus -20, -12, -18 4.87

Left inferior temporal gyrus -52, -2, -34 4.69

Left middle temporal gyrus -60, -14, -16 4.58

*Additional areas of grey matter volume loss in AM at Year 2*

Right anterior inferior temporal cortex

/temporal pole 46, 14, -38 5.11

Right cerebellum 10, -82, -42 3.87

Right temporal pole 24, 10, -36 3.73

Right anterior hippocampus 22, -14, -16 3.66

*Additional areas of grey matter volume loss in AM at Year 3*

Right inferior temporal gyrus 46, 4, -40 4.45


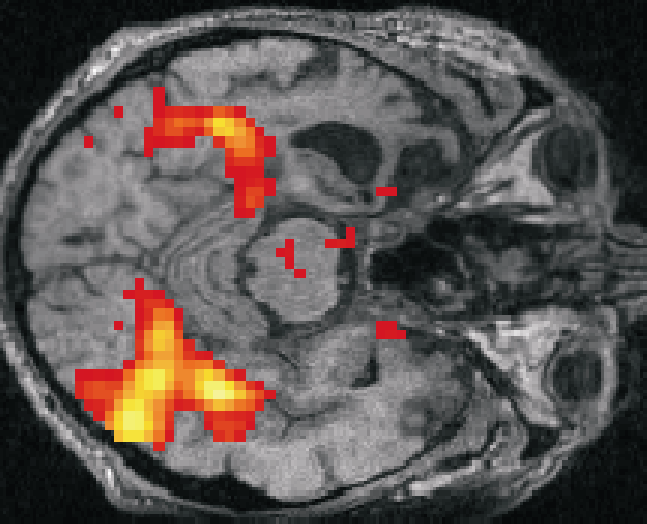


**Fig. S1. Memory for public events in patient AM**

Brain activations associated processing photographs of famous public events compared with the control task (in year 1). Patient AM performed poorly on this task, recognising less than half of the public events, and with only 2/25 events rated as being fully re-experienced. Perhaps unsurprisingly his pattern of posterior occipito-temporal brain responses is similar to those recorded during the processing of unfamiliar foil photographs (see main text Figure 5), because many of the events seemed unfamiliar to AM. Functional images are shown on an axial section from the patient’s structural MRI scan from year 1. Activations are shown at a threshold of p<0.005 uncorrected.


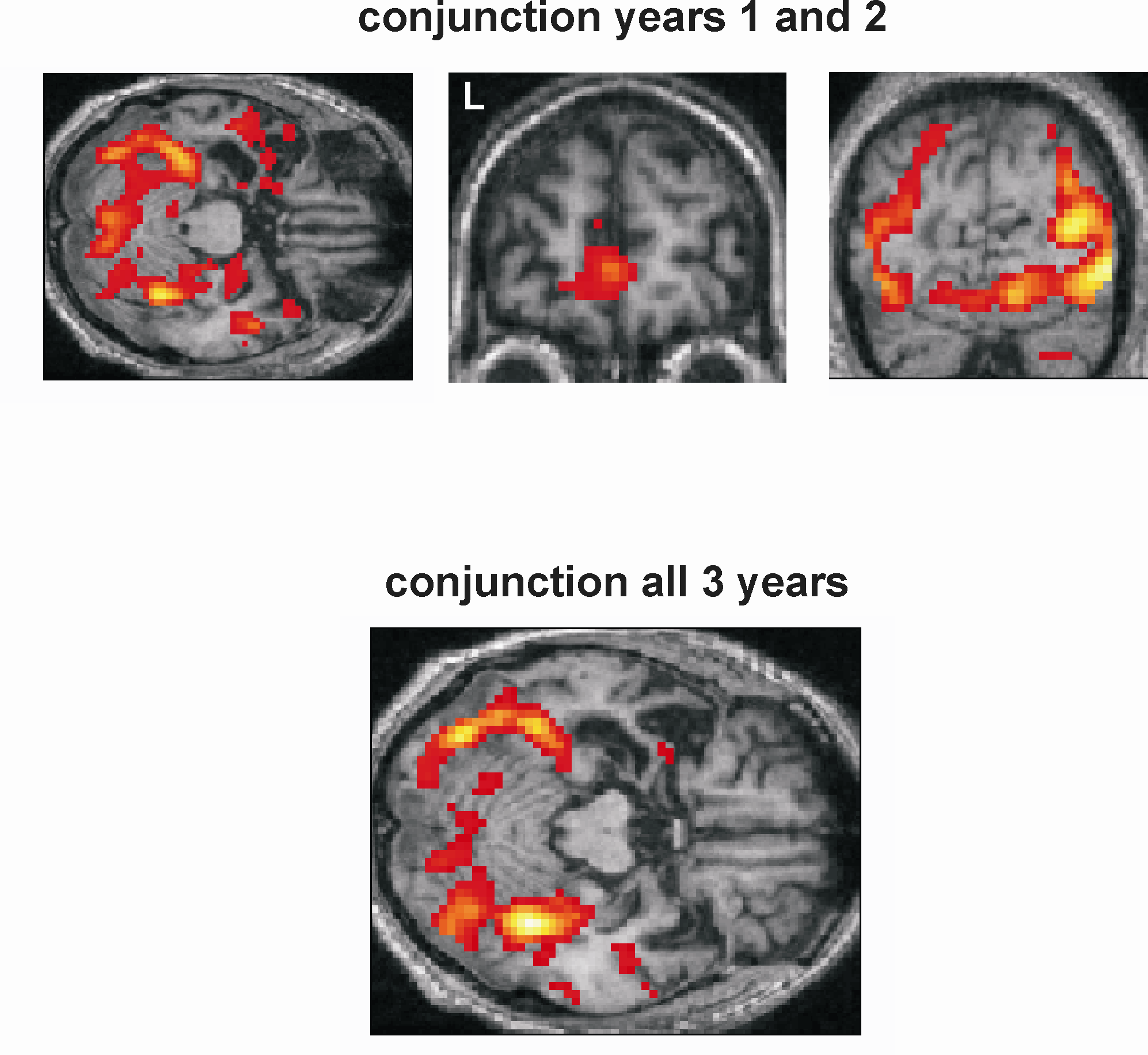


**Fig. S2. Conjunction analyses**

Conjunction analyses show the brain areas that are activated in common for two or more tasks. The top panels show the brain areas that were activated in common for autobiographical memory retrieval in year 1 and year 2 [contrasts in the conjunctions: (memory-control task year 1) (memory-control task year 2)]. Activations are shown on relevant axial, coronal, and sagittal sections from the mean structural MRI scan of the patient averaged across the three years. Activations are shown at a threshold of p<0.005 uncorrected. L=left side of the brain. It is clear that much of the classic autobiographical memory network is active during the two years, including hippocampus, medial frontal and temporal neocortical areas. By contrast, the lower panel shows the conjunction analysis across the three years of the experiment [contrasts in the conjunctions: (memory-control task year 1) (memory-control task year 2) (memory-control task year 3)]. It is clear from this axial view that only posterior occipito-temporal regions are active in common for the three years. This reflects the reduced activity in year 3 of the experiment for autobiographical memory.


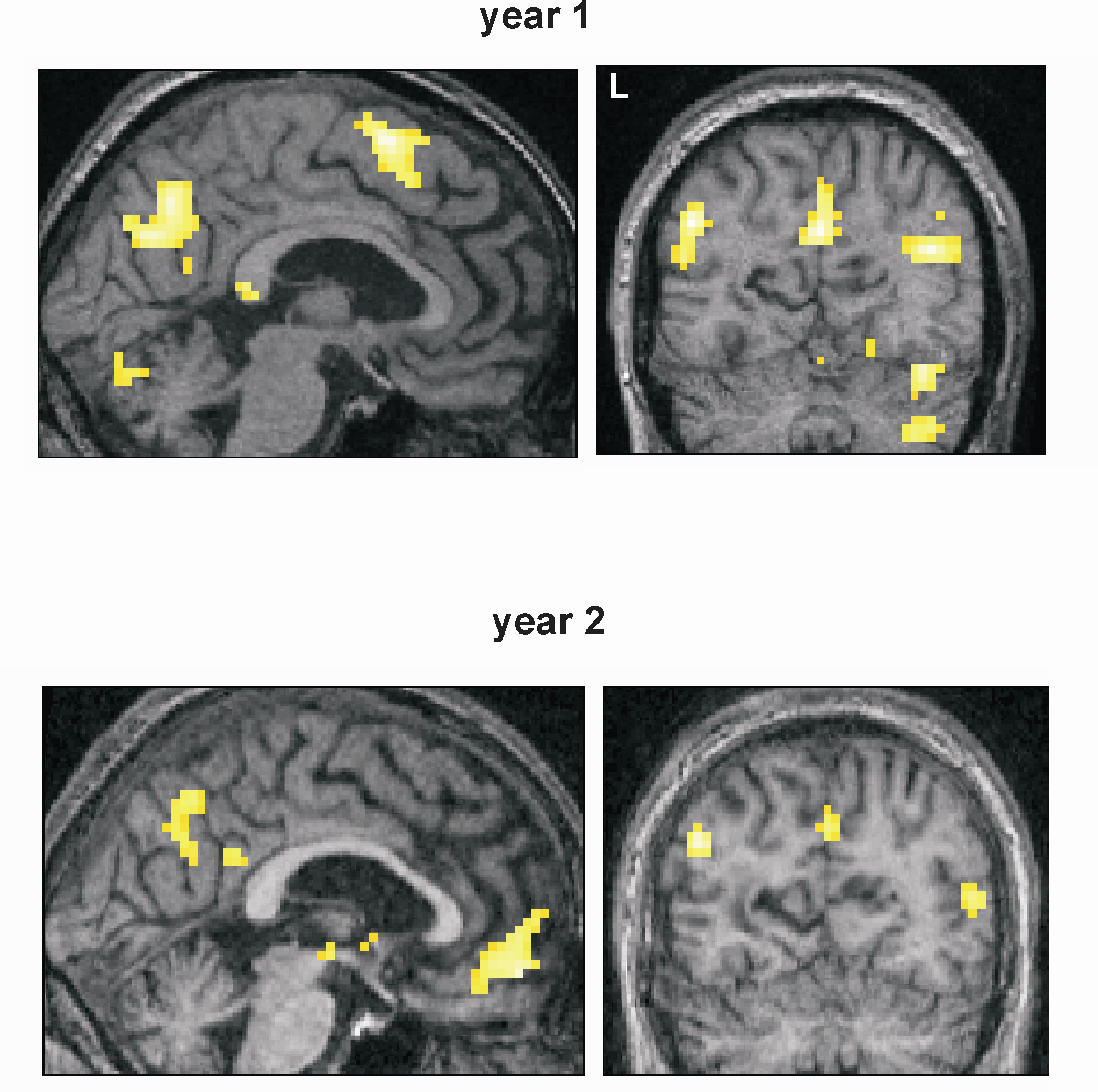


**Fig. S3. Direct comparison of memory and foil photographs**

Brain areas that were more active for autobiographical memory than foil photographs in patient AM were, year 1: superior medial frontal gyrus (-3, 12, 60; Z=5.82); right superior temporal sulcus (39, -72, 21; Z=5.92); left angular gyrus (-45, -72, 30; Z=6.20); precuneus (0, -66, 36; Z=6.17); right cerebellum (36, -69, -24; Z=5.91) (33, -78, -42; Z=5.58). Year 2: ventromedial prefrontal cortex (0, 54, -15; Z=5.84); left angular gyrus (-45, -69, 30; Z=5.77); right temporo-parietal junction (51, -66, 12; Z=5.96); precuneus (0, -69, 36; Z=4.73). Functional images are shown on sagittal and coronal sections from the patient’s structural MRI scan contemporaneous with the functional images for that year. Activations are shown at a threshold of p<0.005 uncorrected. L=left side of the brain. There was no significant difference between autobiographical memory and foil photographs at year 3. The opposite contrast (i.e. foil>memory) showed no significant differences for any year.

Brain areas that were active in this contrast for the control participant were: left precuneus/posterior cingulate cortex (-15, -60,18; Z=5.32); left posterior cingulate cortex (-6, -54,18; Z=4.33); right precuenus (3, -69, 24; Z=3.67); angular gyri (39, -84, 27; Z=5.38; -39, -78, 30; Z=4.12); left and right vmpfc (-6, 33, -6; Z=4.03; 3, 51, -15; Z=3.56); right cerebellum (39, -66, -27; Z=3.91).
